# The retinal determination gene network: from developmental regulator to cancer therapeutic target

**DOI:** 10.18632/oncotarget.9394

**Published:** 2016-05-17

**Authors:** Deguang Kong, Yu Liu, Qian Liu, Na Han, Cuntai Zhang, Richard G. Pestell, Kongming Wu, Gaosong Wu

**Affiliations:** ^1^ Department of Oncology, Tongji Hospital of Tongji Medical College, Huazhang University of Science and Technology, Wuhan, P.R. China; ^2^ Department of Thyroid and Breast Surgery, Tongji Hospital of Tongji Medical College, Huazhang University of Science and Technology, Wuhan, P.R. China; ^3^ Department of Geriatrics, Tongji Hospital of Tongji Medical College, Huazhong University of Science and Technology, Wuhan, P.R. China; ^4^ Department of Cancer Biology, Thomas Jefferson University and Hospital, Philadelphia, PA, USA; ^5^ Department of Medical Oncology, Thomas Jefferson University and Hospital, Philadelphia, PA, USA

**Keywords:** DACH, EYA, SIX, tumor growth, prognosis

## Abstract

Although originally identified for its function in *Drosophila melanogaster* eye specification, the Retinal Determination Gene Network (RDGN) is essential for the development of multiple organs in mammals. The RDGN regulates proliferation, differentiation and autocrine signaling, and interacts with other key signaling pathways. Aberrant expression of RDGN members such as DACH, EYA and SIX contributes to tumor initiation and progression; indeed, the levels of RDGN members are clinically prognostic factors in various cancer types. Stimulation or suppression of the activities of these crucial components can block cancer cell proliferation, prevent cancer stem cell expansion and even reverse the EMT process, thereby attenuating malignant phenotypes. Thus, cancer therapeutic interventions targeting RDGN members should be pursued in future studies.

## INTRODUCTION

Developmental programs control physiological functions at early growth stages, including cell proliferation, differentiation, morphogenesis and tissue homeostasis. Aberrant activation of such programs may disturb the homeostatic balance and thereby trigger tumorigenesis. In this respect, a regulatory network called the *Retinal Determination Gene Network* (RDGN), originally identified as a fundamental signal for *Drosophila melanogaster* eye specification, may also be dysregulated in cancer [1, 2]. Functional modification of the key RDGN members, DACH, EYA and SIX, is a potential therapeutic approach to individualized chemotherapy.

## RDGN SIGNALING, FROM DROSOPHILA EYE DEVELOPMENT TO HUMAN DISEASE

The RDGN first received attention as a key signaling pathway in *Drosophila* eye determination [3]. In the last fifteen years, RDGN signaling has been shown to govern the specification of a wide range of tissues, including the retinas of both insects and mammals. This regulatory network mainly includes a structural relative of the *Ski*/*Sno* gene family, *dachshund*; the SIX family members, sine oculis (*so*) and *optix*; the tyrosine phosphatase, eyes absent (*eya*); and the Pax6 homologs, eyeless (*ey*) and twin of eyeless (*toy*). These genes are organized in a sophisticated network that controls organogenesis, and mutations of these genes in humans are associated with several clinical disorders [3].

The mammalian DACH1 protein can inhibit target gene expression either directly, by binding to specific DNA sequences within chromatin [4, 5], or indirectly, by combining with other transcription factors (c-Jun, SMADs and SIX) [6-9]. For example, DACH1 can displace FOXM1 and FOXC2 from the cis elements within promoters that have Forkhead family binding sites and thereby attenuate their oncogenic activity [4, 5]. During vertebrate development, DACH1 function is required for organ specification [8, 10-12]. In humans, *DACH1* germline mutations have been shown to contribute to bilateral cystic renal dysplasia [13], chronic kidney disease (CKD) [14], familial young-onset diabetes, pre-diabetes and cardiovascular diseases like coronary heart disease (CHD) and coronary arteriosclerosis [15]. DACH1 also inhibits aldosterone secretion in zona glomerulosa cells [16]. However, the functional significance of DACH1 in human diseases still remains a mystery.

As a member of the homeobox gene family, the SIX superfamily has been evolutionarily conserved, and controls embryonic development and tissue specification of the eye, kidney and muscle [2, 3, 17]. During the early stages of development, Six1 activates a diverse range of target genes that determine the proliferation and survival of progenitor cells. Once organ development is complete, Six1 is maintained at low or even undetectable levels in adult tissues [18]. Proteins of the SIX family have two regions of high sequence conservation: the homeoprotein domain (HD) and the SIX domain (SD). SIX proteins recognize specific DNA sequences, and both the HD and the SD contribute to these DNA interactions [19, 20]. Nevertheless, the transcriptional function of SIX depends on an additional cofactor within the complex. For instance, Dach1 functions as a corepressor to inhibit the expression of Six target genes, whereas Eya permits Six to activate downstream signaling [8].

As another component of the conserved RDGN, the EYA family proteins are important transcriptional cofactors. Generally, Eya is recruited to the local chromatin of target genes through Six proteins [8]. Structural analyses have revealed that SIX1 binds to EYA through a single amphipathic helical structure, and that even a single amino acid substitution can abolish SIX1-induced epithelial-mesenchymal transition (EMT) [19]. In humans, mutations in *SIX* and *EYA* or disruption of the SIX/EYA complex cause branchio-oto-rena (BOR) syndrome, an autosomal dominant genetic disorder marked by underdeveloped or absent kidneys, deafness, auricular malformations and bronchial arch remnants [19, 21, 22]. The function of SIX/EYA complex is also required for lung morphogenesis [23] and myocardial hypertrophy [24]. Thus, abnormal functioning of the SIX/EYA complex may impact a broad range of human diseases.

Another critical feature that separates EYA proteins from other RDGN members is their tyrosine/threonine-phosphatase activity. The phosphatase activity of Eya bridges Dach and Six, and switches Six-Dach from a repressed to an activated state by displacing a corepressor and recruiting a coactivator [8]. The conserved carboxy-terminal domain of EYA (ED) is essential for protein-protein interactions, for instance, with SIX and DACH. EYA has been classified into the haloacid dehalogenase (HAD) superfamily based on signature motifs in the ED, and functions as an Mg^2+^-dependent tyrosine phosphatase. The N-terminal region (commonly referred to as ED2), which is characterized by a rich stretch of proline/serine/threonine residues, is mainly responsible for threonine dephosphorylation [2]. This region also provides transactivation domain during *Drosophila* eye development [25]. Because of this phosphorylation mechanism, the EYA family participates significantly in the cellular response to changes in the microenvironment, but the influences of individual family members vary because of their different phosphatase activities. For example, by dephosphorylating H2AX at tyrosine 142 (pY142), EYA1, EYA2 and EYA3 regulate the formation of γH2AX, which promotes DNA repair and cell survival and thus prevents genotoxic stress-induced apoptosis [26, 27]. In the innate immune response, EYA4 serves as a threonine phosphatase rather than as a tyrosine phosphatase [28]. The phosphatase activity of EYA is essential for the maintenance of tight junctions in lung epithelial cells [29]. However, in breast tumors, EYA2 dephosphorylates estrogen receptor β (ERβ) at Y36, thus reversing its antitumor role and promoting malignant growth and dissemination [30]. Likewise, the tumorigenic role of EYA1 in breast cancer cells relies on its phosphatase function [31]. Thus, the phosphatase activity of EYA may be a good target of cancer therapeutics.

**Figure 1:**
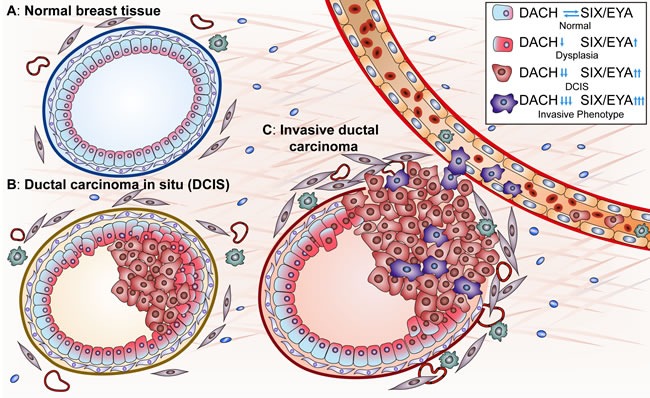
**A.** RDGN members determine breast cancer initiation and progression. In normal breast epithelial cells, the functional balance of DACH and SIX/EYA maintains homeostasis in luminal cellular proliferation/apoptosis and thereby maintains the luminal structure. Functional loss of DACH or hyperactivity of SIX/EYA drives hyper-proliferation, transformation, and progression to ductal carcinoma *in situ*. Some malignant cells undergo EMT, acquire cancer stem cell properties, invade through the basal membrane and enter blood vessels, leading to distant metastases. “↑” represents an upregulated function and “↓” represents a downregulated function.

Recent studies have revealed the significance of the RDGN in tumorigenesis and tumor progression [1, 2]. DACH1 is a tumor suppressor, while SIX/EYA promote malignancy, so aberrations in these components of the RDGN signaling machinery enable cells to acquire oncogenic properties. For example, inactivation of DACH1 or hyperactivation of SIX/EYA in breast epithelial cells leads to over-proliferation, tumor formation and invasion into blood vessels, resulting in distant metastases (Figure 1). These activities of the RDGN members will be discussed in detail below.

## THE RDGN IN TUMOR INITIATION AND PROGRESSION

### Proliferation and cancer stem cells

Constitutive mitogenic signals drive the misexpression of key cell cycle machinery, leading to the initiation and progression of tumorigenesis [32]. The RDGN maintains a dynamic balance that controls progression through the cell cycle. Suppression of cyclin D1 and cyclin A by DACH1 negatively regulates cell cycle progression [33, 34] and thereby blocks cellular proliferation and tumor growth in various cancers [33-35]. In contrast, EYA1 induces cyclin D1 through its phosphatase activity in breast cancer, and SIX1 directly enhances the transcription of *cyclin D1* in rhabdomyosarcoma tumor cells [31, 36]. SIX1 also active cyclin A1 expression to promote proliferation; furthermore, transform mammary epithelial cells and promote aggressive tumor formation and peritumoral lymphovascular invasion [37]. DACH1 promotes cell cycle arrest through context-dependent interactions with p53 and activating p53-taget genes [38, 39], whereas SIX1 downregulates *p53* by upregulating *microRNA-27a-3p* and downregulating ribosomal protein L26 (RPL26) [40]. Imbalance between the tumor-restraining effect of DACH1 and the oncogenic functions of SIX/EYA accelerates cell cycle progression and attenuates apoptosis, creating a favorable environment for uncontrolled proliferation.

Cancer stem cells (CSCs), also called tumor initiation cells (TICs), are characterized by self-renewal and differentiation, and contribute to therapeutic resistance. The regulation of breast CSCs by DACH/EYA/SIX is now well documented. A subpopulation of cells with the CD44^high^/CD24^low^ signature, (where CD44 and CD24 are widely recognized cell surface markers for breast CSCs [41]), can either be negatively regulated by DACH1 as it antagonizes *NANOG*, *KLF4* and *SOX2* transcriptional activation [42], or positively enriched by *SIX1/EYA1* overexpression [43]. SIX1 also activates TGF-β and MAPK signaling to enhance the accumulation of CSCs [43, 44]. In a bitransgenic mouse model, overexpression of human in the adult mouse mammary gland epithelium was found to induce tumors of multiple histological subtypes and to **promote stem/progenitor cell characteristics** by activating Wnt signaling [45]. Similar to SIX1, EYA1 (through its phosphatase activity) allows the proportion of breast CSCs to increase [31]. The therapeutic potential of the RDGN to limit the population of TICs is substantial.

### The RDGN governs EMT and metastasis

**Figure 2:**
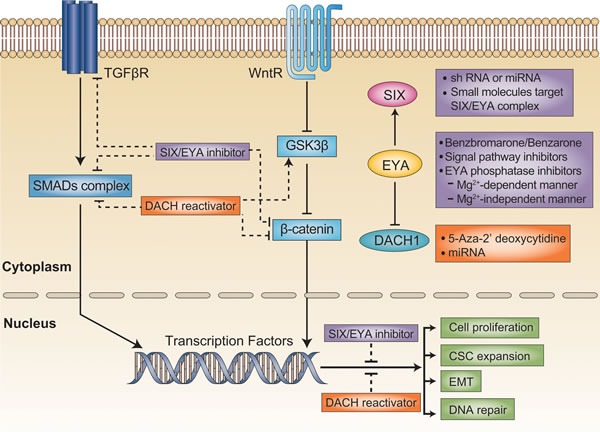
Targeting the DACH/SIX/EYA pathway in cancer treatment TGFβ and Wnt signaling are both critical for tumor initiation and progression. Generally, SIX/EYA activates these two signaling pathways, whereas DACH1 negatively regulates them. The function of SIX could be blocked by the silencing of SIX expression with shRNA, or by the disruption of the SIX/EYA complex with small molecules. EYA phosphatase activity could be blocked by biochemical inhibitors. On the other hand, DACH1 expression could be reactivated with demethylating agents or miRNA. Thus, targeting RDGN members is a promising therapeutic strategy that could restrain malignant behavior in tumors.

EMT, the transformation of epithelial cells into cells with a mesenchymal migratory phenotype, participates in embryonic tissue formation, but also contributes to tumor progression. An interactive network including multiple signaling pathways and post-transcriptional factors facilitates the changes in epithelial cells that drive metastatic seeding [46, 47]. TGF-β-driven cell reprogramming is a multistep process that passes first through EMT. The activation of Wnt signaling creates a favorable environment for TGF-β-induced EMT [48]. The cross-talk between RDGN and Wnt/TGF-β signaling promotes EMT (Figure 2).

DACH1 was first shown to inhibit TGF-β signaling by binding to SMAD4 in breast cancer [49]. Subsequently, DACH1 was found to restrain TGF-β/SMAD-induced EMT [50]. DACH1 may also antagonize EMT in part by inactivating Wnt/β-catenin signaling; indeed, DACH1 was recently found to downregulate β-catenin in hepatocellular and gastric cancer [35, 51]. In contrast, SIX1 promotes EMT in a TGF-β signaling-dependent manner, and is a critical enhancer in the switch of TGF-β/SMAD from tumor suppressors to oncogenic proteins [44, 52, 53]. Even a single amino acid mutation in SIX1 disrupts the SIX1-EYA2 complex, thereby abolishing SIX1-dependent TGF-β signaling and EMT progression [19]. In addition, SIX1-enhanced tumorigenesis is coupled to Wnt signaling activation [53]. EYA2 inactivation reverses the activation of TGF-β/SMAD and EMT induced by SIX1 [54].

The RDGN also governs EMT by regulating the expression of EMT-associated genes. DACH1 was found to abolish YB-1-induced SNAI1 translation and thus reverse EMT in basal-like breast cancer, an aggressive subtype [55]. By downregulating SNAI1, DACH1 increases E-cadherin expression and induces morphological changes that enhance epithelial properties [56]. SIX1 suppresses *miR-200*-family expression, a negative feedback loop in ZEB1/ZEB2 regulation, which in turn promotes EMT and carcinogenesis [57]. The role of EYA in EMT remains to be understood. The tyrosine-phosphatase activity of EYA3 was shown to be critical for cell motility and invasiveness rather than proliferation, indicating that EYA3 promotes metastatic behavior in cancer cells [58]. However, our previous study also indicated that transactivation of cyclin D1 through the tyrosine-phosphatase activity of EYA1 is required for tumor growth and cellular proliferation [31]. Some of these inconsistent results are due to the fact that different researchers have tested the phosphatase activities of the EYAs in different breast cancer cell lines. Although the EYA family members may behave distinctly in different studies, their underlying mechanisms may overlap. Whether they share similar functions during cancer development needs to be further examined.

### The regulation of the tumor microenvironment by RDGN signaling

In response to oncogenic signals from solid tumors or evolving genetic/epigenetic alternations, the tumor microenvironment continually changes to support malignant behavior. Numerous components participate in this process, but cancer-related inflammation and dysregulation of innate immunity especially disturb the physiological homeostasis. It has been indicated that DACH1 binds the endogenous *IL-8* promoter to block breast cancer cellular migration *in vitro* and metastasis *in vivo* [59]. *Dach1* gene deletion in mice was shown to dramatically increase IL-8 and IL-6 abundance by nearly 1000-fold and thus promote prostate cancer cellular migration [60]. CXCL5 is another downstream target whereby DACH1 inhibits lung tumorigenesis and metastasis [61]: endogenous DACH1 negatively regulates CXCL signaling to inhibit cytokine secretion and restrain malignant cell growth and migration. DACH1 also was found to repress bFGF-induced tumor initiation in glioma cells [62]. In contrast, as an enhancer of the innate immune response, EYA4 induces IFN-β and CXCL10 expression by phosphorylating IRF3 and activating NF-κB to against the undigested DNA from apoptotic cells [28]. While the involvement of SIX1 in cytokine secretion and immune regulation is yet to be established, the current evidence suggests that an imbalance in the RDGN would create a favorable microenvironment to enhance the survival of cancer cells and their escape from the primary tumor.

Tumor-associated angiogenesis is another crucial step early in the development and growth of most solid neoplasms, and is also necessary for the hematogenous dissemination of cancer cells. Indeed, EYAs have been detected in vascular endothelial cells and enhance sprouting angiogenesis, mainly by their tyrosine-phosphatase activity [63]. This may explain why transgenic *Eya* knockout mice are characterized by serious defection in the smooth muscle component of the bronchi and pulmonary vessels [64]. Although EYAs promote vascular development, it remains unclear whether they function similarly in endothelial cells and vascular smooth muscle cells.

To invade distant organs, cancer cells must either enter the blood or lymphatic vasculature. The changes in lymphatic vessels can be detected even in early tumor initiation; for instance, the expression of vascular endothelial growth factor C (VEGF-C), a key promoter of lymphangiogenesis, is enhanced at this stage. SIX1 promotes lymphangiogenesis and lymphatic metastasis by upregulating VEGF-C expression [65, 66]. SIX1 transcriptionally activates *VEGF-C* [65], and overexpression of SIX1 in tumor cells may abrogate the inhibitory effect of TGF-β on lymphangiogenesis by upregulating VEGF-C [66]. Considering that EYA2 is an indispensable coactivator in SIX1-induced TGF-β signal activation [19], EYA2 may participate in promoting lymphangiogenesis in a SIX1-dependent manner.

## POTENTIAL STRATEGY OF TARGETING THE RDGN PATHWAY FOR CLINICAL BENEFIT

Successful cancer drugs can be designed against specific molecular targets involved in proliferation, invasion, metastasis and TIC formation [67, 68]. However, discovering novel drug targets is challenging because the inhibition or activation of target genes expressed in normal tissue may result in nonspecific toxicity. Targeting RDGN members like *SIX/EYA*, which are silent or expressed at low levels in adult tissue but are overexpressed in tumors, is a tremendous opportunity for such anticancer therapy. The tyrosine-phosphatase activity of EYA is significant because it promotes DNA damage repair [26] and tumor growth [31], which may promote relapse from chemotherapy and ionizing radiation. Thus, a combination therapy including highly selective EYA inhibitors would reduce the incidence of chemotherapy resistance. Alternatively, reactivation of *DACH1* expression could restrain tumor growth and metastasis [59]. Therefore, therapies co-targeting RDGN members may be more effective and durable cancer treatments than existing treatment options (Figure 2).

### DACH1

Reduced expression of DACH1 tightly correlates with poor prognosis in breast cancer, as was first demonstrated in a tissue microarray analysis of over 2,100 samples, and was subsequently supported in an artificial neural network (ANN) approach [33, 69]. Reduced DACH1 expression also correlates with poor prognosis in prostate cancer and lung and hepatocellular carcinoma patients [35, 59, 70]. Using *Dach1* gene deletion, Dach1 was shown to inhibit prostate cellular proliferation *in vivo* [60]. Still, whereas a number of studies have demonstrated that DACH1 profoundly improves the prognosis of cancer patients as a tumor suppressor, a few studies have also observed an oncogenic role of DACH1 in endometrial cancer and mixed lineage leukemia (MLL) [2, 24, 71]. These seemingly disparate findings might indicate that the exact function of DACH1 depends on its communication with specific signaling pathways, especially in the context of organ heterogeneity.

Various studies have explored the potential of restoring DACH1 expression in cancer cells. Epigenetic silencing of the *DACH1* promoter region is often responsible for its inactivation and the shift towards a malignant phenotypic [50, 51, 72]. Meanwhile, restoration of *DACH1* expression sensitizes gastric and colorectal cancer cells to docetaxel [50, 51] and enhances chemosensitivity to 5-fluorouracil (5-FU) in hepatocellular carcinoma [62]. The next logical step would be to search for a compound that can induce the expression of *DACH1* in tumor tissues. Intriguingly, 5-Aza-2′-deoxycytidine (decitabine), a potent demethylating agent, reactivates *DACH1* in gastric cancer, suggesting that low-dose decitabine combined with traditional chemotherapy may improve the efficiency of treatments against tumors in which *DACH1* expression is silenced [50]. Additionally, ectopic expression of *miR-217* directly inhibits *DACH1* by binding to its 3′UTR, which not only may exhibit the tumorigenic role of *miR-217* in breast cancer, but also suggests an approach for recovering *DACH1* expression by diminishing the activation of a particular miRNA [73].

### SIX1

The current studies have defined an oncogenic role for SIX family members in the development of diverse tumor types. SIX1 combined with specific transcription factors increases a particular subpopulation of cancer stem/progenitor cells. Furthermore, SIX family members are directly responsible for the malignant behavior of cancer cells [2, 37, 53]. Indeed, SIX1 has been highlighted as an independent prognostic marker in colorectal cancer, and its profile can be used to stratify patients into different risk groups and guide individualized regimens [74]. Accumulation of SIX1 reduces paclitaxel sensitivity in patients undergoing breast cancer chemotherapy [75], and diminishes the therapeutic response to tumor necrosis factor-related apoptosis-inducing ligand (TRAIL) in ovarian carcinoma [76]. Intriguingly, overexpression of SIX1 reduces p53 abundance and induces resistance to molecular therapy targeting the murine double mimute2 (MDM2)-p53 interaction. Therefore, the expression of SIX1 could be used as a molecular marker to predict which patients would benefit from MDM2 antagonists [40].

Perhaps the most critical question is how to inhibit the transcription of *SIX1* in a controllable and predictable manner. Silencing *SIX1* expression using short hairpin RNA (shRNA) or miRNA is now viewed as a potential cancer treatment strategy. For instance, *miRNA-185* directly reduces SIX1 abundance and thereby sensitizes SIX1-overexpressing cancer cells to TRAIL-induced apoptosis [77]. *miR-30b* blocks the translation of *SIX1* by binding to its 3′-UTR, thereby delaying the progression of colorectal cancer [78]. Epigenetic modifications, including histone acetylation and methylation, can also restrain the oncogenic role of SIX1 [79, 80]. Since DNA methylation inhibitors (e.g., Dacogen, Vidaza) and broad-spectrum HDAC inhibitors (e.g., Vorinostat, Romidepsin) have been successfully developed, inhibiting epigenetic modifications may be an attractive approach for the multi-modal treatment of advanced cancer [81]. However, it is not known whether these small molecule inhibitors of epigenetic enzymes can block the function of SIX1 *in vivo*.

Although structural and biochemical evidence has confirmed that SIX1 is a DNA-binding transcription factor, the recruitment of additional corepressors or coactivators is necessary for SIX1 signaling [18]. In vertebrates, SIX1 requires EYA for its oncogenic function [19]. Thus, another way to inhibit the aberrant function of SIX1 is by destroying the SIX1/EYA transcriptional complex with a small molecule inhibitor. Since the successful discovery of small molecules like Nutlin-3, which releases p53 from the binding pocket in the MDM2 protein [82], there has been greater hope that effective inhibitors of the SIX1/EYA complex could be generated.

### EYA

EYA proteins facilitate tumor progression and are independent prognostic factors in breast and ovarian cancer [31, 83], but not in pancreatic cancer, in which epigenetic silencing of *EYA2* increases the cancer invasion capacity [84]. The distinct features of the EYA family, whose members can function as either transcriptional coactivators or tyrosine phosphatases, suggest that EYA expression could be inactivated in different ways. EYA activity could be regulated by multiple signaling pathways. Dr. Li and her coworkers demonstrated that hyperactivation of the canonical Wnt and PI3K/Akt signaling pathways reduces EYA1 ubiquitination and thus prevents its premature degradation [85]. In particular, PI3K/Akt signaling was shown to repress the SUMOylation of EYA1 in a phosphorylation-dependent manner and thus enhance the transcriptional activity of EYA1 [86]. Since the knockdown of SUMO-activating enzyme subunit 2 (SAE2) restrains tumor growth and enhances chemosensitivity, it may be possible to suppress EYA expression by promoting its SUMOylation [87]. Meanwhile, combination regimens with specific pathway blockers (PI3K: LY294002; Akt: Perifosine; Wnt: XAV939) may be effective treatments for EYA1-dependent breast cancer.

Several lines of evidence strongly demonstrate that the function of EYA depends on its phosphatase activity; therefore, inhibition of this activity may be another promising treatment strategy. Designing phosphatase inhibitors is challenging, because it requires not only detailed information about the three-dimensional structure of the protein, but also the identification of a unique catalytic property that can be specifically inhibited. In the EYA X-ray crystal structure, aspartic acid appears to be involved in a metal-dependent reaction, rather than the more common cysteine and arginine residues. Thus, EYA is distinct from the classical tyrosine phosphatases in the HAD family, offering a great chance to identify selective EYA phosphatase inhibitors [27, 88, 89]. Seven novel classes of compounds, which bind to the active site of EYA and chelate the active site Mg^2+^ ion, were discovered through structure-based virtual screening technology [90]. Using a structure-based *de novo* design, Dr. Kim's group further identified 29 inhibitors of EYA2 phosphatase with moderate inhibitory activities (IC_50_ values from 6 to 50 μM) [91]. While the tyrosine-phosphatase activity of EYA is critical for regulating the chromatin structure, the threonine-phosphatase activity otherwise determines the involvement of EYA in the innate immune response, and could be inhibited by okadaic acid [28]. However, whether the threonine-phosphatase activity of the EYAs promotes cancer initiation and progression remains to be determined.

Another well-recognized inhibitor of the EYA family is Benzbromarone and its derivative, Benzarone, which selectively blocks both EYA2 and EYA3 activity in a non-competitive way [63, 92]. Pharmacological studies and clinical evaluation in gout treatment have provided comprehensive insights into the safety, metabolism, pharmacokinetics and both chronic and acute toxicity of Benzbromarone and Benzarone. Another advantage of Benzbromarone and its derivatives is the anti-angiogenic effect, which may thereby reduce the neovascular-based dissemination of cancer cells [87]. The major metabolite of Benzbromarone, 6-hydroxy Benzbromarone, is a more powerful inhibitor of EYA tyrosine-phosphatase activity, and may block tumor growth by inhibiting angiogenesis [92]. As a result of these studies, there is now a discussion of whether luminal B-type breast cancer patients could benefit from Benzbromarone-based combination therapy due to its suppression of EYA [31].

Recently, a fluorescent high-throughput-screening phosphatase assay identified a series of N-arylidenebenzohydrazide-containing compounds that inhibit the catalytic site of EYA2 compared with other cellular phosphatases in HAD family [93]. These compounds selectively inhibit the phosphatase activity of EYA2 rather than EYA3 [94]. In contrast with those metal-chelating compounds and Benzbromarone, which physiologically perturbs metal ions, N-arylidenebenzohydrazide-containing compounds bind to the allosteric site in a Mg^2+^-independent manner [89]. Since EYA2-induced migration could be reversed by these compounds, EYA2 phosphatase-specific anti-cancer drugs appear promising. With great technological advancements, like the establishment of high-throughput material screening platforms, it will be possible to find a specific EYA inhibitor for clinical application.

## CONCLUSION AND PERSPECTIVES

The RDGN could directly or indirectly determine the biological features and clinical outcomes of various cancer types, so RDGN genes are promising biomarkers. Evaluation of profiles of RDGN members could guide the design of individualized treatment regimens [95]. At the core of this research area are the questions: Which particular cancers are driven by the RDGN? And then, how will it be possible to specifically reactivate the expression of DACH1 or efficiently block the functions of SIX1/EYA? At this time, only a few drugs and small molecules have been shown to counteract the aberrant expression of RDGN members *in vitro*. Therapeutic agents targeting the RDGN are worthy of development in light of the profound biological effects of these gene products in cancer progression and metastasis.
